# Risk perception and transmission potential of *Neospora caninum* at the wildlife and livestock interface in Minnesota

**DOI:** 10.3389/fvets.2025.1552390

**Published:** 2025-03-06

**Authors:** Larissa A. Minicucci, Michelle Carstensen, Louis Cornicelli, Stacey A. Elmore, Jitender P. Dubey, Paul Wolf, Erik Hildebrand, Devin Tunseth

**Affiliations:** ^1^College of Veterinary Medicine, University of Minnesota, St. Paul, MN, United States; ^2^Minnesota Department of Natural Resources, Wildlife Health Program, Forest Lake, MN, United States; ^3^Animal Parasitic Diseases Laboratory, Beltsville Agricultural Research Center, United States Department of Agriculture, Agricultural Research Service, Beltsville, MD, United States; ^4^United States Department of Agriculture, Animal and Plant Health Inspection Service, Wildlife Services, St. Paul, MN, United States

**Keywords:** *Neospora caninum*, dogs, cattle, wolf, transmission, domestic, risk, canid

## Abstract

Neosporosis is a major cause of abortion in cattle with significant economic consequences for infected farms. We collected sympatric human dimensions, livestock, and wildlife data in a pilot study to assess the understanding and significance of *Neospora caninum* on Minnesota cattle farms and address the biases of producers who often implicate wolves (*Canis lupus*) for exposing cattle to this parasite. We surveyed veterinarians and producers to assess their knowledge and attitudes regarding *N. caninum*. We also conducted on-farm risk assessments and estimated *N. caninum* seroprevalence in domestic and wild animals. Our survey work showed that producers lack an understanding regarding neosporosis and an overall gap in communication exists between veterinarians and their clients relative to risks associated with *Neospora*. Overall seroprevalence for *N. caninum* on 10 farms (7 beef, 3 dairy) was 20.9% (*n* = 450 cattle tested), with individual herd seroprevalence ranging from 0 to 51.3% (median = 9.1%; mean = 16.4%, std. = 19.0%). We found no difference in seroprevalence of *N. caninum* between farms within and outside of wolf range. Seroprevalence among domestic canid samples was 64.3% (9/14) and among felid samples was 25% (5/20); most farms had at least one seropositive dog and cat. Most farms (90%) had at least one wildlife species test seropositive for *N. caninum*. On farm risk assessments, combined with serological data, provided strong evidence that domestic dogs present the greatest risk for exposure of *N. caninum* to cattle. Enhanced communication between veterinarians and producers can foster better outcomes by proactively reducing risk of disease transmission and accepting their role in the outcomes.

## 1 Introduction

*Neospora caninum* is a protozoal parasite that is best known for causing abortion in cattle and neurologic disease in dogs (*Canis familiaris*). Dogs, coyotes (*Canis latrans*), and wolves (*Canis lupus*) are its definitive host where the sexual cycle occurs, leading to excretion of environmentally resistant oocysts in feces. Numerous domestic and wild animals are its intermediate host. Both domestic and sylvatic transmission cycles occur. In Minnesota, the sylvatic cycle is characterized by transmission between cervids (e.g., white-tailed deer [*Odocoileus virginianus*] and moose [*Alces alces*] (1–3)) and wild canids (i.e., gray wolves and coyotes) (4–6), whereas domestic *N. caninum* cycles occur between livestock (e.g., cattle, sheep, and goats) and dogs (7). Canid definitive hosts gain exposure by eating infected animals, placental tissue, or fetuses. Numerous domestic and wild animal species can serve as intermediate hosts. Also, transplacental infection from mother to fetus is especially common in cattle and perpetuates neosporosis within a herd (7). Consequently, overlap between the sylvatic and domestic cycles of *N. caninum* transmission is an ongoing concern for animal health workers and livestock producers worldwide.

In northern Minnesota, approximately 80% of white-tailed deer are infected with *N. caninum* (2), and as the primary prey for wolves, deer provide continued opportunity for wolf exposure (8). Carstensen et al. (9) reported that *N. caninum* antibodies were detected in 61% (173/285) of wolves sampled throughout their range in Minnesota (2009–2013). Also, Gondim et al. (1) found *N. caninum* antibodies in 39% (64/164) of free-ranging gray wolves (Minnesota), 11% (12/113) of coyotes (Utah, Colorado, and Illinois), 26% (50/193) of white-tailed deer (Minnesota and Illinois), and 13% (8/61) of moose (Minnesota). These data support a sylvatic transmission cycle of *N. caninum* between cervids and canids. The authors speculated that hunting by humans also favored the transmission of *N. caninum* from cervids to canids, because cervid carcasses are usually eviscerated in the field and scavenged by wildlife.

Previous research has shown that at least half the dairy and beef herds in the United States have one or more animals that have been exposed to *N. caninum* (7). In an infected herd, up to 30 percent of the animals may test positive, and some cows may abort for several consecutive pregnancies (10, 11). While the cow shows little to no effect of the parasite, it can be lethal to fetuses (12, 13). Transplacental transmission (i.e., vertical transmission) is the primary mode of transmission of the parasite within cattle herds and although surviving heifers from seropositive cows might be asymptomatic, they could still pass the parasite on to their own calves, or be more likely to abort fetuses (7). The role of horizontal transmission, however, should not be discounted because this is how *N. caninum* may enter a herd. Both canids and livestock can also gain exposure by consuming contaminated feed or water, grazing on contaminated pastures, or by scavenging contaminated tissue (i.e., horizontal transmission). This disease is one of the major reasons farm dogs should not eat aborted fetuses, fetal membranes, or dead calves; doing so may increase the risk of oocyst exposure to livestock. Neosporosis is a major problem in dry lot dairies where feed at the central feed storage could be contaminated with *N. caninum* oocysts. Breeding beef cattle in USA are often raised on open range pasture and exposure is more sporadic. Even if the rangeland contains canid scat and *N. caninum* oocysts, the oocysts will not be concentrated at a central feeding location (7).

The estimated economic impact of neosporosis within U.S. dairy industry is at least $546 million, annually (14). On an infected farm, several factors contribute to the economic burden including abortions, stillbirths and neonatal mortality, infertility, increased culling, decreased milk production, and decreased value of breeding stock (14–16). In acutely affected herds, abortion rates as high as 57% have been reported (17), translating to a significant potential economic loss. In the absence of a suitable vaccine, the current control strategies rely on disrupting the parasite life cycle and have their own associated cost. For example, producers with infected herds could purchase replacement cows from naïve farms rather than using home-bred daughters of seropositive cows. Although this might increase costs for the producer, it could help to disrupt the cycle of vertical transmission within the female bloodline. Other control actions include serologically testing replacement cows, embryo transfer to prevent transplacental transfer of the parasite, and artificial insemination from serologically negative bulls (7). Also, protecting fodder from oocyst contamination by farm dogs and other canids is important, because this is a common route by which a naïve herd is exposed to *N. caninum* (7).

Minnesota cattle producers are concerned about wolf presence near farms relative to risk of calf depredation and disease transmission, including the potential challenges associated with *N. caninum* exposure (18). Cattle producers often perceive a higher risk of infection for facilities within wolf geographic range than those on the outside. As wolf management remains a politically charged issue with management authorities shifting repeatedly between federal and state governments in recent decades, there is increased interest in how new management strategies may impact disease incidence. Also, the epidemiological roles of other, more common, wildlife (and domestic) species frequently observed on farms remains incomplete. Informed scientific data is needed to better answer these questions to appropriately inform policymaking.

The primary goal of this pilot study was to assess the understanding of sylvatic and domestic neosporosis in Minnesota. Although the surveys and farm histories included information about *N. caninum* transmission from cow to calf, this study focused on horizontal transmission of the parasite through oocysts (canids as definitive hosts) or carnivory (animals feeding on infected tissue). Specifically, we sought to determine: (1) the knowledge and attitudes held by cattle producers and large animal veterinarians regarding neosporosis, (2) the prevalence of *N. caninum* within selected Minnesota cattle herds located within and outside of wolf range, and (3) the prevalence of *N. caninum* in canids and other common wildlife species inhabiting these farms.

## 2 Methods

### 2.1 Producer surveys

A 27-question survey (S1) was designed for cattle producers to assess disease knowledge and management and prevention factors related to *N. caninu*m. The producer survey was determined to be exempt from review by the University of Minnesota Institutional Review Board (IRB) (HSC 1401E46882). A list of dairy producers in Minnesota was obtained from the Minnesota Department of Agriculture (*n* = 3,771) and a list of beef producers (*n* = 18,543) was obtained from the Minnesota Board of Animal Health via a public data request. Producers were categorized into locations based on whether they were within or outside of wolf range based on the wolf range map.^1^ One thousand farms were randomly selected using the RAND function in Microsoft Excel (software information) from the beef producer list (*n* = 500 farms) and the dairy list (*n* = 500 farms) respectively, with 250 in each category representing wolf territory and 250 representing non-wolf territory. Surveys were mailed to the 1,000 producers identified via random selection and reminder postcards were sent four weeks after distribution of the original survey.

### 2.2 Veterinarian surveys

A 24-question survey (S2) was designed for large animal veterinarians to assess disease knowledge and management and prevention factors related to *N. caninu*m. The veterinarian survey was determined to be exempt from review by the University of Minnesota IRB (IRB HSC 1401E46882). Contact information for mixed and large animal veterinarians (*n* = 354) in the state of Minnesota was obtained from the Minnesota Veterinary Medical Association. Additionally, a list of members (*n* = 113) of the American Association of Bovine Practitioners (AABP) working in Minnesota was obtained from the AABP after project review. Surveys were mailed to all 467 veterinarians identified, and a follow up was not conducted.

### 2.3 On-farm risk assessment

Following an extensive literature review, an on-farm risk assessment (S3) was developed to evaluate multiple aspects on a cattle farm that may predispose it to neosporosis infection. Areas evaluated included demographics and location, general farm characteristics, facilities and management, biosecurity (including evaluation of dog access and wildlife) and herd health. Ten key risk categories based on the assessment were scored as ‘low’, ‘moderate’, or ‘high’ risks and communicated as feedback to producers. The risk assessment was determined to be exempt from review by the University of Minnesota IRB (IRB HSC 1401E46882).

### 2.4 Farm selection process

**Figure 1:**
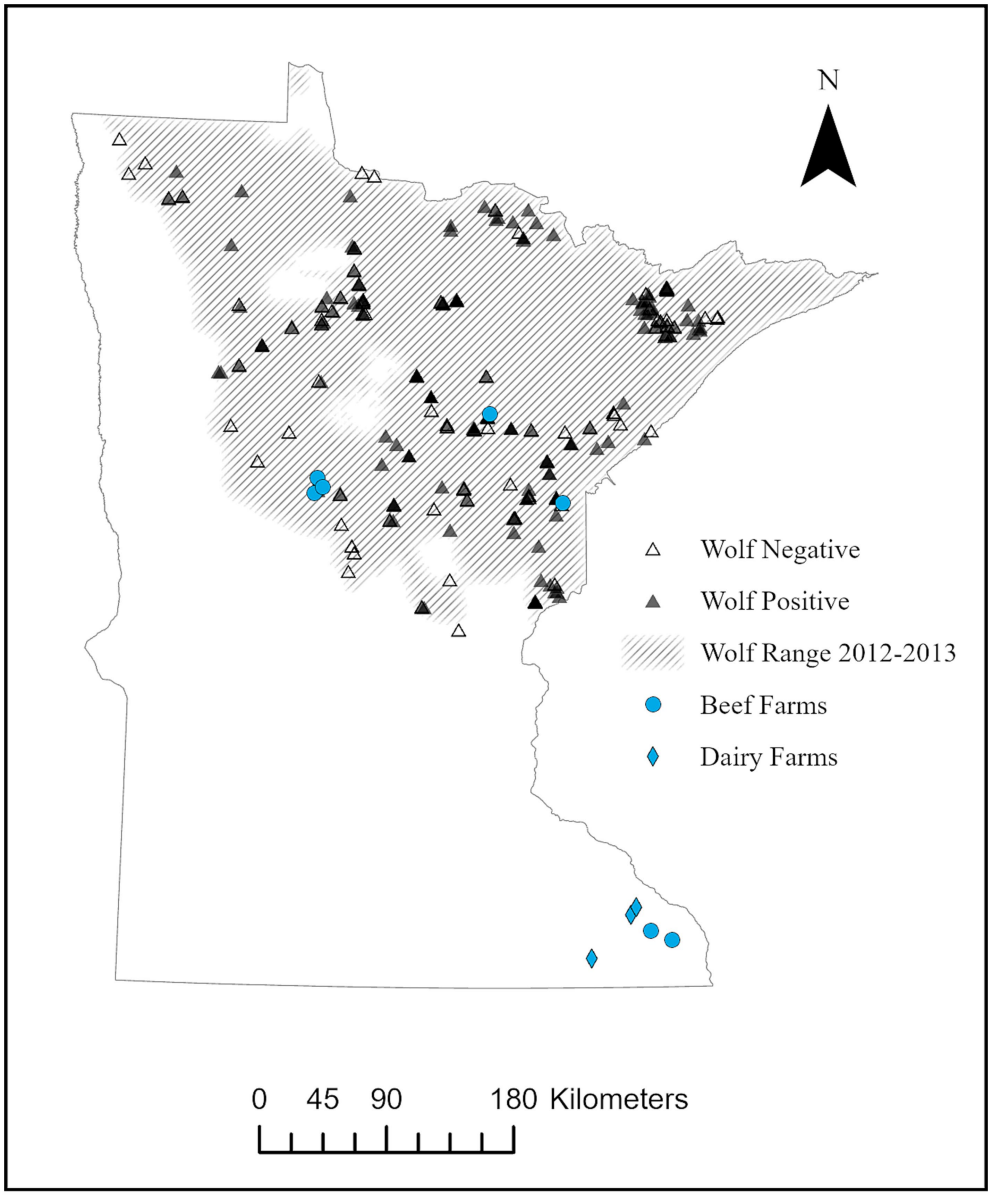
Location of beef (*n* = 7) and dairy (*n* = 3) farms participating in pilot study for serologic exposure to *Neospora caninum* in cattle, domestic animals, and local wildlife from August – December 2014, relative to wolf (*Canis lupus*) range in Minnesota. Locations of wolves found positive (*n* = 173) or negative (*n* = 112) during a previous serosurvey of exposure to *N. caninum* across wolf range from 2010–2013 (8) is also included.

Ten farms throughout Minnesota were identified as pilot sites, including both northern farms inside (*n* = 5) and southern farms outside (*n* = 5) of known wolf range (Figure 1). The ten farm sites were chosen after reviewing abortion and *N. caninum* serology data within the University of Minnesota Veterinary Diagnostic Laboratory (UMVDL) database; farms with previous neosporosis diagnoses, herds with histories of abortions, or known livestock-wildlife conflict were targeted (19). The estimated density of wolves within wolf range was 2,423 (95% CI = 1,935–2,949 or 3.4 wolves/100 km^2^) in 2014 (20). A serologic survey of wolf exposure to *N. caninum* occurred across wolf range from 2010–2013 and 173 of 285 (61%) were positive (Figure 1) (9). Coyotes range throughout Minnesota with an estimated density of 14,490–28,980 or 22–33 coyotes/100 km^2^ (21). No prior information was available on coyote exposure to *N. caninum* across Minnesota.

### 2.5 Domestic animal sample collection and testing

Study workers visited each farm once to collect blood samples from the coccygeal vein of cattle and the cephalic or lateral saphenous vein of domestic dogs. Additional samples were collected opportunistically from dogs (feces) and cats (blood from a jugular vein) on each site. Cattle sera were tested for *N. caninum* antibodies at the (UMVDL) using a commercially available enzyme-linked immunosorbent assay (ELISA; Idexx Laboratories, part number 99–09566). Fecal samples were submitted to UMVDL for flotation using a double centrifugation technique with zinc sulfate medium (specific gravity: 1.18) to screen for *N. caninum-*like oocysts.

### 2.6 Wild animal sample collection and testing

Minnesota Department of Natural Resources (MNDNR) contracted with the United States Department of Agriculture, Animal Plant and Health Inspection Service, Wildlife Services (USDA/APHIS/WS) for wild animal trapping and euthanasia on farms (Special Permit No. 18926). Wildlife trapping was conducted for four trap-nights at each of the 10 farm sites utilizing a combination of foothold (Duke #3 – Coil Spring with Offset Jaw, West Point, MS) and cage traps (Havahart – Large 2-Door Animal Trap, Lancaster, Pennsylvania, USA). Trapping sites were selected through conversations with landowners about known wildlife activities, species commonly observed, and trail cameras placed in select locations to assist in identifying species presence. The wildlife species identified on farms included raccoon (*Procyon lotor*), striped skunk (*Mephitis mephitis*), coyote, gray fox (*Urocyon cinereoargenteus*), bobcat (*Lynx rufus*), opossum (*Didelphis virginiana*), Eastern cottontail rabbit (*Sylvilagus floridanus*), red fox (*Vulpes vulpes*), groundhog (*Marmota monax*) and American badger (*Taxidea taxus*). Wolves were not targeted during this study as previous serological data on exposure to *N. caninum* were available (9) and state management did not authorize additional take. Traps were checked daily during the trapping window and animals were humanely euthanized via gunshot with a small caliber firearm (IACUC Protocol 1302-30345A). Blood samples were collected from each animal post-mortem, centrifuged within 12 h of collection, and serum decanted into cryovials and stored at −20°C. The dog, cat, and wildlife sera were tested at the United States Department of Agriculture, Agricultural Research Service, Animal Parasitic Diseases Laboratory (Beltsville, Maryland) for *N. caninum* antibodies by *Neospora caninum* agglutination test (NAT) (22). Sera were diluted 2-fold 1:25 to 1:100 and positive and negative controls were included in each test. Fecal samples were submitted to UMVDL for flotation using a double centrifugation technique with zinc sulfate (specific gravity: 1.18) to screen for *N. caninum-*like oocysts. Additional samples (e.g., brain and heart) were collected from each animal for a companion study on exposure to *Toxoplasma gondii* (23).

### 2.7 Data analysis

Producer and veterinarian survey data were analyzed using the Statistical Package for Social Sciences, Version 22 (SPSS; IBM Corp., Armonk, N.Y., USA). We calculated frequencies to assess attitudes toward and knowledge about *N. caninum* for both survey groups. We performed a chi-square test to assess differences among producer respondents living within, on the fringe, or outside wolf range; results were considered significant at *p* < 0.05. Finally, seroprevalence for cattle, domestic animals, and wildlife was determined by dividing the number of positive animals by the number of animals tested.

## 3 Results

### 3.1 Producer surveys

One hundred thirty-five surveys were completed and 28 were not deliverable, yielding a response rate of 13.9% (135/972). The distribution of responses is summarized as follows: beef/wolf range = 35 (28.9%), beef/outside wolf range = 29 (23.97%), dairy/wolf range = 18 (14.95), and dairy/outside wolf range = 39 (32.2%).

Overall, 29% (*n* = 38) of respondents had heard of *Neospora* and there were no differences among people who lived inside (28%), on the fringe (adjacent county; 40%), or outside (22%) Minnesota wolf range (χ^2^ = 3.251, *p =* 0.119). Producers first learned about the disease most often from their veterinarian (43%), followed by a magazine/book (41%), or another producer (22%). They were least likely to learn about the disease from the internet (19%) or conference (3%). Slightly more than one-third correctly identified the disease as being caused by a parasite (36%); other respondents incorrectly identified the disease as being cause by a bacteria (17%), virus (8%), or fungus (6%); 33% did not know the causative agent. The majority (58%) did not know how common the disease was in their area; similarly, a plurality (42%) also did not know how important the disease was to cattle. However, all respondents believed *Neospora* was not a risk to humans, although only 12% had spoken with their veterinarian about the disease.

Most respondents (*n* = 97; 74%) owned a domestic dog, and nearly all dogs (97%) had access to cattle pasture and barns. Overall, 69% indicated that wildlife other than rodents and small birds had contact with cattle. The most frequently listed species were deer (75%), birds (50%), and coyotes (48%). Wolves were noted by 40% of respondents who lived either in wolf range or the fringe. Regarding modifying farming practices if *Neospora* was discovered, about half (55%) were undecided; however, 41% would be either somewhat (24%) or very likely (17%) to modify farming practices.

### 3.2 Veterinarian surveys

One hundred twenty-four (of 462) surveys were completed and five were not deliverable, yielding a response rate of 26.8%. Overall, 77% of responding veterinarians practiced outside the established wolf range, and 80% had been practicing for at least 10 years. As expected, nearly all respondents (98%) had some familiarity with the disease; however, only 41% had diagnosed it on a farm. Most frequently reported symptoms were abortion (98%) and poor reproductive performance (38%). Regarding how common and important *Neospora* is in the area they practice; we observed no statistical differences for respondents within and outside the wolf range. A plurality of respondents personally believed the disease was uncommon (41%) and slightly important (36%). Respondents within the wolf range were slightly more likely to discuss the disease with producers, although it was rarely discussed (63% in wolf range, 44% outside). When asked about how their clients felt about the disease, 43% of veterinary respondents indicated they believed it was not important.

To minimize risks of disease introduction and spread, respondents recommended keeping domestic dogs out (56%), maintaining a clean feeding area (34%), and removing seropositive cattle (18%). Biosecurity recommendations centered on feed protection (68%), removing dead stock (55%), and good sanitation practices (48%). Wildlife control by lethal removal was noted by 43% of respondents as a method to reduce risk.

### 3.3 Cattle seroprevalence

**Figure 2:**
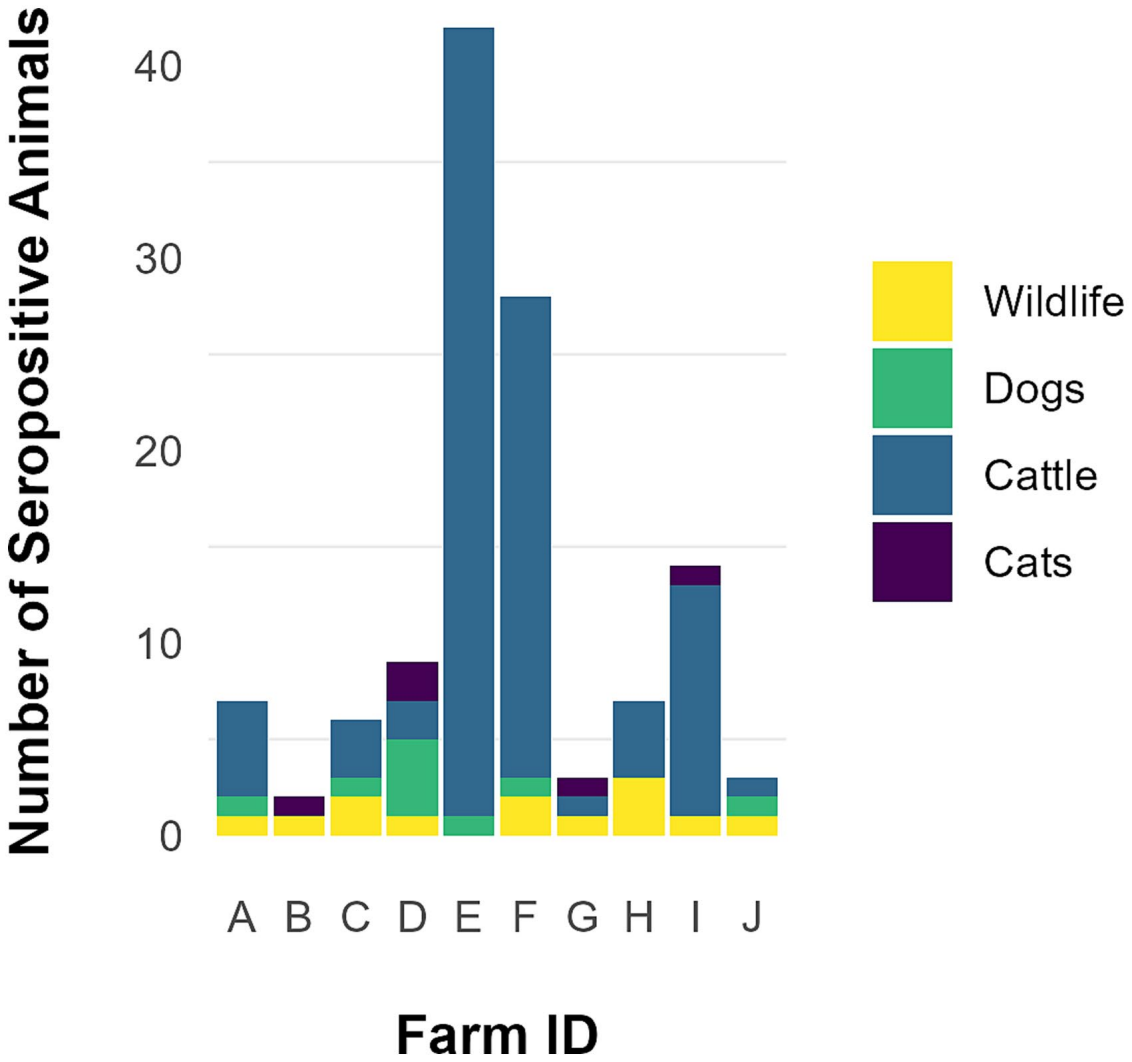
Total animals, including wildlife (*n* = 13), dogs (*n* = 9), cats (*n* = 5), and cattle (*n* = 94) found seropositive for *Neospora caninum* on 10 farms participating in a pilot study from August–December 2014 in Minnesota.

Cattle herd sizes (*n* = 10) ranged from 32 to 1,382 animals (median = 534.3; mean = 524.8, std. = 390.8). Beef cattle herd (*n* = 7) sizes ranged from 32 to 1,382 animals (median = 548.5; mean = 496.5, std. = 466.0) and dairy cattle herd (*n* = 3) sizes ranged from 475 to 770 animals (median = 520; mean = 590.7, std. = 162.9). A total of 450 cattle from the 10 farms were sampled for *N. caninum* exposure (Table 1; Figure 2). Overall seroprevalence for *N. caninum* was 20.9% (94/450), with individual herd seroprevalence ranging from 0 to 51.3% (median = 9.1%; mean = 16.4%, std. = 19.0%). Overall seroprevalence for beef animals was 18.9% (56/297), with individual herd seroprevalence ranging from 0 to 51.3% (median = 7.1%; mean = 12.7%, std. = 17.3%; Table 1). Overall seroprevalence for dairy animals was 24.9% (38/153), with individual herd seroprevalence ranging from 2 to 50% (median = 22.6%; mean = 24.9%, std. =24.1%; Table 1). Northern county animals (inside wolf range) had a cumulative seroprevalence of 21.3% and individual herd seroprevalence ranging from 0 to 51.3% (median = 6.3%; mean = 14.5%, std. = 20.8%); southern county animals (outside of wolf range) had a cumulative seroprevalence of 20.4% and individual herd seroprevalence ranging from 2 to 50% (median = 9.1%; mean = 18.2%, std. = 19.4%; Table 1). Using the chi-square test for goodness of fit, no statistically significant difference was noted between the number of infected cattle in northern counties versus southern counties (OR = 1.06, *p*-value = 0.8).

**Table 1:** *Neospora caninum* seroprevalence of cattle (*n* = 450) by farm location and cattle type, sampled during August–December 2014 in Minnesota.

Farm ID	Location	Cattle type	Samples collected (*n*)	Positive samples (*n*)	Seroprevalence (%)	Standard error	95% Confidence interval
A	North	Beef	54	5	9.3	0.04	(0.02, 0.17)
B	North	Beef	22	0	0.0	0.00	(0.00, 0.00)
C	North	Beef	51	3	5.9	0.03	(0.00, 0.12)
D	North	Beef	32	2	6.3	0.04	(0.00. 0.15)
E	North	Beef	80	41	51.3	0.06	(0.40, 0.62)
F	South	Dairy	50	25	50.0	0.07	(0.36, 0.64)
G	South	Beef	14	1	7.1	0.07	(0.00, 0.21)
H	South	Beef	44	4	9.1	0.04	(0.01, 0.18)
I	South	Dairy	53	12	22.6	0.06	(0.11, 0.34)
J	South	Dairy	50	1	2.0	0.02	(0.00, 0.06)

### 3.4 Other domestic animals seroprevalence and fecal analysis

Sera from 14 domestic dogs and 20 domestic cats from nine different farms were evaluated for *N. caninum* exposure (Table 2; Figure 2). Seroprevalence among canine samples was 64.3% (9/14) and among feline samples was 25% (5/20); 55.6% (5/9) and 44.4% (4/9) of farms had at least one seropositive dog and cat, respectively (Table 2). A total of 10 fecal samples collected from dogs on six different farms, and 13 fecal samples collected from domestic cats on eight different farms were evaluated for *N. caninum* oocysts. *Neospora caninum*-like oocysts were not detected in any domestic animal fecal samples.

**Table 2:** *Neospora caninum* seroprevalence of domestic canines and felines present on farms by location and cattle type, sampled during August–December 2014 in Minnesota.

Farm ID	Location	Cattle type	Canine sera collected (*n*) and seroprevalence (%)	Feline sera collected (*n*) and seroprevalence (%)	Overall domestic animal seroprevalence (%)
A	North	Beef	1 (100)	1 (0)	50.0
B	North	Beef	2 (0)	4 (25)	16.7
C	North	Beef	1 (100)	5 (0)	16.7
D	North	Beef	4 (100)	2 (100)	100.0
E	North	Beef	1 (100)	1 (0)	50.0
F	South	Dairy	1 (100)	0	100.0
G	South	Beef	1 (0)	2 (50)	33.3
H	South	Beef	1 (0)	1 (0)	0.0
I	South	Dairy	0	3 (33)	33.3
J	South	Dairy	2 (50)	1 (0)	33.3

### 3.5 Wildlife seroprevalence and fecal analysis

Forty-one wild animals from 10 different farms were trapped and sera tested for *N. caninum* exposure (Table 3; Figure 2). Thirteen of 41 (31.7%) samples were seropositive with distribution as follows: 1/1 coyote, 1/1 red fox, 2/2 Eastern cottontails, 1/1 bobcat, 1/2 badgers, 1/3 groundhogs, 5/22 raccoons, 1/7 striped skunks, 0/1 Virginia opossums, and 0/1 gray fox. Nine (90%) farms had at least one wildlife serum sample that was *N. caninum* positive. Of the three wild canids trapped on three different farms, two (66.7%) were seropositive (Table 3). *Neospora caninum*-like oocysts were not detected in any wildlife fecal samples.

**Table 3:** *Neospora caninum* seroprevalence of wildlife (*n* = 41) by species (*n* = 10) trapped on farms during August–December 2014 in Minnesota.

Farm ID	Total wildlife sera collected (*n*)	Positive sera by wildlife species^1^	Overall wildlife seroprevalence (%)
A	4	1/1 rac, 0/2 sku, 0/1 gra	25.0
B	2	1/1 sku, 0/1 rac	50.0
C	7	1/1 coy, 1/2 rac, 0/4 sku	28.6
D	3	1/1 bob, 0/2 rac	33.3
E	1	0/1 rac	0.0
F	3	1/2 bad, 1/1 rab	66.6
G	3	1/1 rac, 0/2 ghg	33.3
H	6	1/1 red, 1/1 rab, 1/1 ghg, 0/2 rac, 0/1 opo	50.0
I	11	1/11 rac	9.1
J	1	1/1 rac	100.0

^1rac, raccoon; sku, striped skunk; gra, gray fox; coy, coyote; bob, bobcat; bad, badger; rab, Eastern cottontail; ghg, ground hog; red, red fox; opo, Virginia opossum.^

### 3.6 Risk assessment

Seven of 10 farms scored moderate risk for feed storage (outside, unfenced, covered; outside, fenced uncovered; or in an open building). The remaining three farms scored high risk (feed stored outside, unfenced, uncovered) (Table 4). Five of 10 farms scored low risk for water source (potable well water or public water supply) and four of 10 farms scored high risk (stream, lakes, ponds, etc.). The remaining farm scored moderate risk for water source (non-potable well water). All 10 farms were considered high risk related to their *N. caninum* testing protocols (either no testing performed, or testing of only cows that abort and *N. caninum*-positive cattle are not removed from the herd). Seven of 10 farms scored high-risk regarding disposal of placentas, aborted tissue, and deadstock (left out in the open). Two of 10 farms scored moderate risk (tissues removed and buried to prevent predation or composted but still accessible to animals and wildlife). The remaining farm scored low risk regarding disposal of placentas, aborted tissue and deadstock (composted in a fenced area and/or removed from premises) (Table 4). Seven of 10 farms scored low risk for cattle source as they only introduced bulls to the herd for breeding purposes. One farm scored high risk as the producer introduces heifers of unknown *N. caninum* and reproductive status. Two farms had completely closed herds.

**Table 4:** Risk assessment rankings relative to *Neospora caninum* exposure by farm, August–December 2014, in Minnesota.

Farm ID	Feed storage	Water source	*Neospora caninum* Testing Protocol	Disposal of placentas, aborted tissues, deadstock	Cattle Source	Dogs on farm	Wild canids on farm	Use of isolation pens for birthing	History of abortion
A	High	High	High	Low	Low	Moderate	High	Low	High
B	High	High	High	High	High	High	High	Moderate	Low
C	High	High	High	High	Low	Moderate	High	Low	High
D	Moderate	High	High	High	Low	High	High	Low	High
E	Moderate	Low	High	High	Low	High	High	Low	High
F	Moderate	Low	High	Moderate	n/a^1^	High	High	High	High
G	Moderate	Low	High	High	Low	High	High	Moderate	Moderate
H	Moderate	Low	High	High	Low	Moderate	High	Low	High
I	Moderate	Low	High	Moderate	Low	Low	Moderate	Low	High
J	Moderate	Moderate	High	High	n/a^1^	High	Moderate	Low	High

^1n/a, close herd.^

Six of 10 farms scored high risk for dogs on premises (dogs have free or occasional access to cattle housing and/or feed storage areas) and three of 10 farms scored moderate risk for dogs on premises (dogs located on premises but have no access to cattle housing and/or feed storage areas) (Table 4). The remaining farm scored low risk, as no dogs were allowed on the premises. Eight of 10 farms scored high risk for wild canids on farm property (wild canids are frequently seen on the premises in cattle housing and/or feed storage areas) and two of 10 farms scored moderate risk as wild canids are seen on the premises but have no access to cattle housing and/or feed storage areas.

Seven of 10 farms never used isolation or birthing pens and scored low risk for this category (Table 4). Two of 10 farms fell into the moderate risk category for occasional use of isolation pens as birthing pens, and one farm was considered high risk for frequent use of birthing pens as isolating pens. Eight of 10 farms scored high risk for either a history of abortions attributed to *N. caninum* or to an unknown cause and one farm scored moderate risk due to a history of abortions from a known cause other than *N. caninum.* The remaining farm scored low risk because of no known history of abortions on the farm (Table 4).

## 4 Discussion

This study examined *N. caninum* ecology holistically in which we collected sympatric human dimensions, livestock, and wildlife data, albeit at a small scale. Although neosporosis is a serious disease of cattle, our survey work shows a communication and education gap in how veterinarians and producers perceive and understand the disease. For example, nearly all veterinarians were familiar with *Neospora* and 41% had even diagnosed it on a farm, yet nearly half felt this disease was not important to their clients and few producers ever spoke to their veterinarian about it. Veterinarians also recommended keeping domestic canids away from cattle; however, most producers owned a dog that had direct access to cattle. Survey results were validated by the on-farm risk assessments, which also demonstrated domestic dog access to cattle. There was also a lack of belief in disease importance from producers or a willingness to implement control measures, which may be an educational opportunity on the part of veterinarians when they are interacting with their clients. Given the potential adverse economic consequences of neosporosis, it was interesting that producers were largely undecided if they would make changes even if the disease was discovered on their farm. However, it is costly for producers to submit aborted fetal and placental tissue to diagnostic laboratories (more than $100 [USD]) and control mechanisms may be expensive or difficult to implement. As a result, interest in neosporosis research has declined (but the problem has not). Although there is no current estimate for neosporosis-driven economic loss in the United States, the annual worldwide estimate from a decade ago was at least $1.3-billion-dollars (USD) (7).

Neosporosis continues to be major cause of abortion in cattle worldwide, particularly dairy cattle; *N. caninum* was identified in as many as 20% of abortions (7). The neosporosis burden in Minnesota is not well estimated, but from 1991 to 2011, *N. caninum* caused 4.5% of the abortion cases submitted to the University of Minnesota Veterinary Diagnostic Laboratory (18). The economic losses are associated primarily with vertical transmission and subsequent neonatal mortality, as adult cattle infected with *N. caninum* are asymptomatic and cow-to-cow transmission is not known to occur (7). However, horizontal transmission via exposure to oocysts is also important because this is the route by which *N. caninum* can be introduced to a naïve herd or maintained in a herd through ongoing exposure (7). Seroprevalence of *N. caninum* varies with the management, type of cattle, serological tests used and the locality. In the USA, there are very few serological surveys, and most information is from dry lot dairies in California (17, 24, 25). In one survey of 2,585 cows in 55 beef herds in five northwestern states of USA (Idaho, Montana, Oregon, Washington, Wyoming), mean *N. caninum* seroprevalence, using a commercial ELISA was 23% (26). In a relatively large dairy herd in eastern USA (Maryland) *N. caninum* antibodies were detected in 28% of 1,029 cattle, using a high cutoff value (1,200), in an indirect fluorescent antibody test (27). In the present study, antibodies to *N. caninum* were found in 21% of cattle by using a commercial ELISA test. To our knowledge, this is the first serosurvey of cattle herds for *N. caninum* antibodies conducted in Minnesota, which might explain why many producers lacked awareness of local occurrence.

Historically, northern Minnesota contained both dairy and beef farms; however, the feasibility of the feasibility of maintaining northern milk truck routes declined in the 1980s and dairies became less common (E. Mousel, pers. comm). Today, dairies and feedlots are concentrated in southern Minnesota where producers can better access commerce centers, major transportation routes, and can also grow feed crops (e.g., corn) (E. Mousel, per comm). Alternatively, northern Minnesota, is more likely to host cow-calf operations in the beef industry; these cattle spend more time grazing over rangeland and wooded areas (E. Mousel, pers. comm). In southern Minnesota, beef farms often run both cow-calf and feedlot operations. While *N. caninum* infection is not a main concern for feedlot cattle, the breeding stock might be affected and have higher risk (E. Mousel, pers. comm). Even if producers maintain cow-calf stock by rotational pasture grazing, they might also give supplemental feed from the feedlot and become exposed to *N. caninum* through feed contamination (E. Mousel, pers. comm). Dairies, like feedlot operations, will feed their stock from a concentrated feed source rather than depending on open-range grazing. In the present study, two of the 3 dairies (southern Minnesota) are considered closed herds; however, all of the dairies were found to have seropositive wildlife, cattle, and domestic animals. This finding supports the recommendation that all cattle producers routinely screen their stock for *N. caninum* antibodies and send any aborted tissue to diagnostic labs for testing. Simply having a closed herd does not protect against neosporosis because the parasite can be introduced through a concentrated feed source instead of new stock. By definition, producers with closed herds are breeding their own replacement heifers but if they are not testing for *N. caninum*, the parasite could go unnoticed until it starts affecting a significant portion of the herd; this could be very costly (7).

Cattle, dogs, and wildlife all demonstrated seropositivity on six farms and the other four farms had seropositive individuals in two groups out of the three (i.e., cattle/dog: E, dog/wildlife: B, and cattle/wildlife: H and I). Given this mixture and the proximity among sampled individuals, unquantified overlap between the domestic and sylvatic transmission cycles might also exist. The wildlife present on these farms had substantial *N. caninum* seropositivity (31.7% of 41 total samples), again providing evidence for sylvatic neosporosis. Among Minnesota wildlife, wolves and coyotes are the only known definitive host species (4–6, 28) and can potentially distribute oocysts throughout the environment. However, we did not detect *N. caninum* in any canid (wild and domestic) fecal samples during this study and wolves were not targeted for sampling. Although more work is needed to describe the concentration and duration of oocyst shedding by wild definitive hosts, the existing information from experimental inoculations and snapshot fecal analyses suggest that most definitive hosts only shed oocysts briefly and in low numbers (7). This is unlike the related parasite, *Toxoplasma gondii*, where cats excrete millions of oocysts. When *N. caninum*-like oocysts are detected by fecal flotation, this finding must be confirmed with additional diagnostics (e.g., polymerase chain reaction, bioassay). *Hammondia heydorni* and *H. hammondi* oocysts are morphologically identical and more common in canid feces (7). Bioassay is the only true confirmatory test and we did not pursue molecular work on feces when no oocysts were identified morphologically. One of the co-authors of the present study (JPD) pioneered research on *N. caninum* oocysts from wild canids; in that work, oocysts were identified in wolf feces by flotation in sucrose solution (specific gravity 1.15) and then confirmed by bioassay in gamma interferon gene knock out mice (6). Future research should evaluate how both domestic and wild canids contribute to environmental contamination of *N. caninum* oocysts across a landscape and how this influences transmission risk to cattle herds.

Of the 10 wildlife species sampled in this study, only coyotes are recognized definitive hosts of *N. caninum* (5). One red fox and one bobcat were also seropositive; neither foxes nor felids are demonstrated definitive hosts of *N. caninum*. The remaining nine seropositive species might be intermediate hosts of the parasite, thus serving as reservoirs and increasing the landscape distribution through movement (e.g., dispersal and routine space-use). Further, both wild and domestic canids would prey or scavenge upon nearly all the wildlife species we sampled on the farms (provided there was opportunity to do so), and their infected tissues could contribute to maintenance of *N. caninum* and ongoing exposure risk. The role of peri-domestic birds and small rodents in *N. caninum* ecology, if any, was not explored in this study; however, these animals might be important non-ungulate sources of *N. caninum* exposure if infected individuals (e.g., with tissue cysts) are eaten by farm dogs or wild canids near farms (29, 30) Both types of animals draw canids to feed storage areas and if infected canids happen to be shedding oocysts, feed contamination could occur (7). Therefore, the control of peri-domestic birds and rodents could be examined in future studies on these dual-operation farms.

Our survey results do not indicate that veterinarians and producer views of neosporosis are correlated with the presence of wolves, nor do our serological results indicate that wolves play a significant role in *N. caninum* epidemiology on Minnesota cattle farms. The cumulative cattle seroprevalence between northern and southern farms were similar (21.3 and 20.4%, respectively); if wolves drive the domestic transmission of farm-side *N. caninum*, we expect the cattle seroprevalence on northern farms to be consistently higher than on southern farms. Coyotes might have a larger role than wolves because they have a statewide distribution, are more abundant, and consistently exist along rural–urban landscape gradients (31, 32). The most likely explanation is that domestic dogs are the key definitive canid host on cattle farms in Minnesota (7). This represents a potential challenge for producers that want to minimize risk of *N. caninum* transmission on farms, which may entail restricting access of farm dogs to cattle, pastures, and most certainly aborted tissues and deadstock, or opting to not have dogs at all. Overall, while wolf-associated neosporosis risk is geographically restricted to Minnesota beef farms, coyotes and domestic dogs might be implicated in neosporosis risk to both industries.

Our pilot study highlights the need for additional education for veterinarians and producers alike regarding risk mitigation and enhanced biosecurity practices to safeguard cattle from *Neospora caninum* exposure from multiple potential pathways. Neosporosis is a complex disease and future research could use longitudinal data to better understand the seasonality and other temporal characteristics that affect transmission dynamics. Additional follow-up to this pilot study could explore regional differences in *Neospora* epidemiology, especially in areas with contrasting farm management and wildlife communities. Our broad approach to this topic, while limited in sample size, shows the complexity of communicating risks of disease transmission at the wildlife-domestic animal interface. Often it may be more palatable for producers to focus on risks outside of their direct control (e.g., presence of various wildlife species on the landscape, near farms) than those they can influence, such as behavior and access of dogs to livestock. Enhanced communication between veterinarians and producers can foster better outcomes by proactively reducing risk of disease transmission and accepting their role in the outcomes.

## Data Availability

The raw data supporting the conclusions of this article will be made available by the authors, without undue reservation.

## Footnotes

^1^ https://files.dnr.state.mn.us/wildlife/wolves/wolf-plan.pdf
