# Supplementary material for: Risk perception and transmission potential of Neospora caninum at the wildlife and livestock interface in Minnesota
Source: Front Vet Sci. 2025 Mar 6;12:1552390. doi: 10.3389/fvets.2025.1552390 (PMC11924202; doi:10.3389/fvets.2025.1552390)
Supplement: Supplementary file 1 [file Data_Sheet_1.pdf]

UNIVERSITY OF MINNESOTA

CENTER FOR ANIMAL HEALTH
AND FOOD SAFETY

Neosporosis: Producer Knowledge and Attitudes Survey

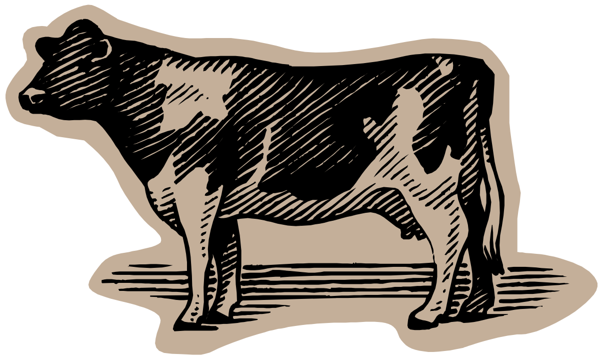

University of Minnesota – Center for Animal Health and Food Safety

Principal Investigator: Larissa Minicucci, DVM, MPH, DACVPM

Neosporosis: Producer Knowledge and Attitudes Survey

Thank you in advance for taking the time to complete this survey. Your participation is very important and will help increase our knowledge of Neospora in Minnesota and our understanding of how you, as a cattle producer, manage this important disease.

All survey participants who return a completed survey will be entered into a drawing to receive one of three $50 gift cards to Cabela's.

Section A: General Demographics

County where cattle are located: ___________________________

Please check the 'Yes' box for each type of operation you own and also indicate the number of cattle on the farm for each type of operation.

Type of Operation	Yes	Number of cattle on farm
Beef cow-calf		
Stocker /Backgrounder		
Feedlot/ Farmer Feeder		
Dairy (milking)		
Dairy (heifer raiser)		
Other: ________________________________		

What type of housing system is used for cattle? Please circle all that apply.

a. Seasonally pasture based

b. Open Lot

c. Confinement Building

d. Barn (please specify) Tie Stall ☐ Free Stall ☐

e. Other (please specify)____

Section B: Disease Knowledge

1. **Have you heard of Neosporosis or Neospora *(Neospora caninum)* before? Please circle one.**

   Yes No (If no, please skip to Section C)

2. **If "Yes", where did you first hear of Neospora Please check all that apply.**

   ____ Another producer
   ____ Veterinarian
   ____ Magazine/Book (specify): ________________________________
   ____ Conference (specify) ____________________________________
   ____ Internet
   ____ Other (specify):____

3. **Have you ever had Neospora on your farm? Please circle one.** Yes No

4. **If "Yes", briefly describe the signs/symptoms experienced by the animal(s)/herd:**

   ____

5. **Have other producers near your farm (within 10 miles) had Neospora diagnosed on their farm? Please circle one.**

   Yes No

   If yes, how many producers? #___________

6. **Do you know any other producers (besides those within 10 miles of your farm) in Minnesota that have had Neospora on their farm? Please circle one. (If no, please skip to question 8)**

Yes No

a. If yes, how many producers? #___________
b. If yes, where in Minnesota (list counties)? ____________________________

7. **What months did you see signs/symptoms of Neospora?** (Please circle all that apply)

January	April	July	October
February	May	August	November
March	June	September	December

8. **How common do you think Neospora is in your area? Please check one.**

__Very Rare __ Uncommon __ Very Common
__ Rare __ Common __ Don't Know

9. **How important of a problem is Neospora in your area? Please check one.**

__ Unimportant __ Neutral __ Very Important
__ Slightly Important __ Important __ Don't Know

10. **Which animals do you think can get sick from Neospora? Please check all that apply.**

____ Cattle ____ Deer
____ Pigs ____ Coyotes
____ Horses ____ Wolves
____ Sheep/Goats ____ Foxes
____ Poultry ____ Raccoons
____ Dogs ____ Skunks
____ Cats
____ Other (specify): ____

11. **Which animals do you think are carriers of Neospora? Please check all that apply.**

____ Cattle ____ Deer
____ Pigs ____ Coyotes
____ Horses ____ Wolves
____ Sheep/Goats ____ Foxes
____ Poultry ____ Raccoons
____ Dogs ____ Skunks
____ Cats
____ Other (specify): ____________________________________

12. **In your opinion, which one animal (wild or domestic) is the single most important carrier of Neospora**? ____

13. **What kind of an organism do you think Neospora is? Please check one.**

__ Bacteria __Parasite __Don't Know
__ Virus __Fungus __Other (Please specify)___________________________

14. **In your opinion what is the most important way that Neospora can be spread to cattle?**
____
____
____

15. **Do you think Neospora is a health risk to humans? Please circle one.** Yes No

Section C: Management and Prevention

16. **Do you keep a dog(s) on the farm? Please circle one.** Yes No

17. **If "Yes", do they have access to the pasture/barns? Please circle one.** Yes No

18. **Other than rodents and small birds, do you frequently have wildlife on your property that come into contact with cattle? Please circle one.** Yes No

19. **If "Yes" to question 18, briefly list wildlife species that come into contact with your cattle:**

____
____
____

20. **How are you currently managing wildlife on your farm? Please check all that apply.**

____ Bait
____ Shooting
____ Other (Specify):____________
____ Trapping
____ Fencing
____ Nothing

21. **If you currently use fencing on your premises, please answer the following questions.**

i. What type of fencing is used on the farm? Please circle all that apply.

a. Barbed Wire
b. Electrified wire
c. Combination barbed wire/electrified
d. Woven wire
e. Other: ___________________________________

ii. What is the minimum fence height (perimeter fence) on the farm?

a. Less than 6 feet
b. 6 – 8 feet
c. More than 8 feet

iii. What is the primary function of fencing on the farm? Please circle one.

a. Keeping cattle contained
b. Keeping deer off property
c. Keeping other wildlife off property
d. Preventing trespassing
e. Other:______________________________

22. **Are you currently utilizing any prevention practices for Neospora in your cattle? Please circle one.**

Yes No

23. **If "Yes", which prevention practices do you use? Please check all that apply.**

____ Vaccination
____ Medication
____ Blood Testing
____ Other (Specify): _______________________________________

24. **Have you ever spoken with your veterinarian, diagnostic laboratory, or extension agent about Neospora? Please circle one.** Yes No

25. **If "Yes" to question 24, briefly describe what they recommended**. ____________________
____
____
____
____

26. **If Neospora were/was found on your farm or in the area, how willing would you be/were you to make changes to your farming practices (such as putting up fences, setting up testing protocols, etc.). Please circle one number.**

Not at all Willing		Undecided		Very Willing
1	2	3	4	5

27. **If you have made changes to your farming practices because of Neospora, please explain what changes you have made.** ____
____
____
____
____

Other comments: ____
____

Thank you for your participation!
