# Supplementary material for: Risk perception and transmission potential of Neospora caninum at the wildlife and livestock interface in Minnesota
Source: Front Vet Sci. 2025 Mar 6;12:1552390. doi: 10.3389/fvets.2025.1552390 (PMC11924202; doi:10.3389/fvets.2025.1552390)
Supplement: Supplementary file 2 [file Data_Sheet_2.pdf]

Other comments: ____

____

____

____

____

Thank you for your participation!

UNIVERSITY OF MINNESOTA

CENTER FOR ANIMAL HEALTH AND FOOD SAFETY

Neosporosis: Veterinarian Knowledge and Attitudes Survey

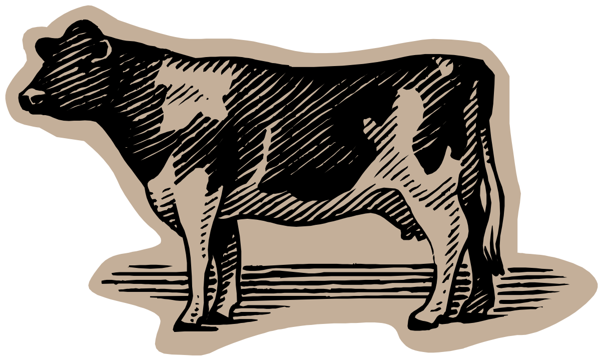

University of Minnesota – Center for Animal Health and Food Safety

Principal Investigator: Larissa Minicucci, DVM, MPH, DACVPM

Neosporosis: Veterinarian Knowledge and Attitudes Survey

Thank you in advance for taking the time to complete this survey. Your participation is very important and will help increase our knowledge of Neospora in Minnesota and our understanding of how you, the practicing veterinarian, manage this important disease.

All survey participants who return a completed survey will be entered into a drawing to receive one of three $50 gift cards to Cabela's.

Section A: General Demographics

Please list all counties you practice in: ______________________

____

____

Type of Practice (Please circle only one):

1. Food Animal
2. Mixed Animal (>50% food animal and/or equine)
3. Mixed Animal (<50% food animal and/or equine)
4. Small Animal
5. Equine
6. Other (Please specify): ______________________________

Have you ever worked with food animals as a licensed veterinarian? Please circle one. Yes No

How many years have you been practicing veterinary medicine?

____

What percent of your clients raise beef or dairy cattle?

________________% Dairy

________________% Beef

❑ <-- Check here if you do not work with cattle

Section B: Disease Knowledge

In this section, we are interested in learning about your knowledge and familiarity with Neospora. Please fill out this section even if your practice is exclusively small animal and/or you've never practiced in a large animal clinic.

1. **How familiar are you with *Neospora caninum* (Neosporosis)? Please check one.**

__ Not at all Familiar __ Moderately Familiar

__ Slightly Familiar __ Very Familiar __ Extremely Familiar

2. Where have you learned about Neospora? Please check all that apply.

__ Primary literature

__ Conference

__ University

__ Another veterinarian

__ A client

__ Internet

__ Other (Please specify): ______________________________

3. Have you ever diagnosed Neospora on a farm in your current practice? Please check one.

__ Yes

__ No <-- Please skip to Question 8

4. How many total farms have you diagnosed with Neospora:

________Farms

5. Indicate the signs/symptoms experienced by the animal(s)/herd. Please check all that apply.

__ Abortion

__ Poor Reproductive Performance

__ Decreased Production

__ Neurologic signs in calves

__ Weak calves

__ Other (Please specify): __________________________

6. What diagnostic tests have you used to diagnose Neospora on the affected farm(s)? Please check all that apply.

__ IHC

__ PCR

__ Serum ELISA

__ Fecal Test

__ Other (Please specify): ______________________________

__ None, I have never done a Neospora test

7. What month(s) did you see signs/symptoms of Neospora? Please circle all that apply.

January March May July September November

February April June August October December

8. How common do you think Neospora is in the area(s) you practice veterinary medicine? Please check one.

__Very Rare __ Uncommon __ Very Common
__ Rare __ Common __ Don't Know

9. How important of a problem do <u>you</u> believe Neospora is in your area? Please check one.

__ Unimportant __ Neutral __ Very Important
__ Slightly Important __ Important __ Don't Know

10. How important of a problem do cattle producers you work with think Neospora is in your area? Please check one.

__ Unimportant __ Neutral __ Very Important
__ Slightly Important __ Important __ Don't Know

11. In your opinion, which animals can develop clinical illness as a result of Neospora? Please check all that apply.

__ Cattle __ Deer
__ Pigs __ Coyotes
__ Horses __ Wolves
__ Sheep/Goats __ Foxes
__ Poultry __ Raccoons
__ Dogs __ Skunks
__ Cats
__ Other (Please specify): ______________________

12. In your opinion, which animals are carriers of Neospora? Please check all that apply.

__ Cattle __ Deer
__ Pigs __ Coyotes
__ Horses __ Wolves
__ Sheep/Goats __ Foxes
__ Poultry __ Raccoons
__ Dogs __ Skunks
__ Cats
__ Other (Please specify): _________________________

13. In your opinion, which one animal (wild or domestic) is the single most important carrier of Neospora?

____

____

14. Which diagnostic tests would you prefer to use to diagnose Neospora? Please check all that apply.

__ IHC
__ PCR
__ Serum ELISA
__ Fecal Test
__ Other (specify):________________

15. Please describe what you think is the most important way that *Neospora* can be transmitted to cattle:

____

____

____

____

____

<u>Section C: Management and Prevention</u>

<u>If you work exclusively in a small animal clinic, please do not answer these questions. Your survey is complete. Please return it in the self-addressed business reply envelope.</u>

16. How frequently do you discuss Neospora with cattle producers who are your clients? Please check one.

__ Never	__ Sometimes	__ Always
__ Rarely	__ Often	__ Not Applicable

17. What prevention practices for Neospora do you recommend to your clients? Please check all that apply.

__ Vaccination

__ Antibiotics/Antiparasitics

__ Blood Testing

__ Removal of Neospora seropositive cattle

__ Other (Please specify): ____________________________

__ None

18. What biosecurity practices do you recommend to your producers for Neospora prevention/management? Please check all that apply.

__ Wildlife control (e.g., hunting and trapping)

__ Fencing

__ Sanitation

__ Protection of feedstuffs

__ Removal of dead stock and placentas from farm

__ Dietary management

__ Other (Please specify): ___________________________

__ None

19. How willing are the producers you work with to follow your recommendations? Please check one.

__ Never	__ Sometimes	__ Always
__ Rarely	__ Often	__ Not Applicable

20. What practices are producers most willing to implement in response to Neospora? _______________

____

____

____

____

____

21. What practices are producers least willing to implement in response to Neospora? _________________________

____

____

____

____

____

22. What reasons do cattle producers cite for not following your recommendations? __________________________

____

____

____

____

____

23. Which resources would you use to get more information about Neospora?

__ Primary literature

__ Lay publications

__ Textbook

__ District veterinarian

__ Minnesota Board of Animal Health

__ U.S. Department of Agriculture (USDA)

__ Internet

__ Other

24. Which resources do you direct clients to regarding Neospora management and control? ________________

____

____

____

____

____
