# Supplementary material for: Risk perception and transmission potential of Neospora caninum at the wildlife and livestock interface in Minnesota
Source: Front Vet Sci. 2025 Mar 6;12:1552390. doi: 10.3389/fvets.2025.1552390 (PMC11924202; doi:10.3389/fvets.2025.1552390)
Supplement: Supplementary file 3 [file Data_Sheet_3.pdf]

University of Minnesota
CENTER FOR ANIMAL HEALTH
AND FOOD SAFETY

Neosporosis on the Minnesota landscape

Instructions: This risk assessment form is to be used to evaluate cattle operations to determine potential risk of *Neospora caninum* transmission at the livestock and wildlife interface. If cattle are kept at multiple sites, please complete an additional risk assessment form for each site.

Date: ____________________

Survey Conducted by: ____________________________

Name of Farm Owner: ____

Mailing Address: ____

City: ____________________________ **Zip:** ________________ **Phone:** _________________

Premises ID: _________________ **National Premises ID:** ____________________________

County: _____________________ **Township:** _____________ **Range:** _________________

Section: ____________________ **Latitude:** N____________ **Longitude:** W ___________

Total acreage (own and/or lease): ____________

Additional cattle locations (please circle)**:** Yes No (If yes, complete additional form)

Cattle Address: ____

County: _____________________ **Township:** _____________ **Range:** _________________

Section: ____________________ **Latitude:** N____________ **Longitude:** W ___________

Section A: General Farm Characteristics

1. Type of operation? Circle all that apply.

1. Beef cow-calf
2. Beef feeding
3. Dairy cattle
4. Dairy heifer raiser
5. Other (please specify) ________

2. Number of cattle?

1. Adult beef cattle_______
2. Beef youngstock_______
3. Adult dairy cattle_______
4. Dairy youngstock_______

3. What breed(s) of cattle are present?

____

____

4. Number of other livestock on premises?

1. Sheep________
2. Goats________
3. Cervids (captive)________
4. Camelids________
5. Swine___________
6. Horses__________
7. Poultry _________
8. Other___________

5. **Number of farm workers? __________**

6. **How many acres do you farm? ___________**

    a. **How much of that land is designated for cattle? ____________**

7. **Land type around main farm operation** (please write in %):
    1. Crop Fields__________%
    2. Upland Hardwoods_________%
    3. Lowland Swamp__________%
    4. Pasture___________%
    5. Other (please describe): ____%

8. **What is the primary water source used for your cattle?**
    a. Stream, lake, or pond
    b. Well water (potable)
    c. Well water (non-potable)
    d. Public water supply

Section B: Facilities and Management

9. **What type of housing/production system is used for cattle?**
    a. Extensive, pasture based
    b. Part pasture, part barn
    c. All barn   Tie Stall ☐   Free Stall ☐
    d. Other (please specify)__________________

10. **If cattle are on pasture, is rotational grazing used?**
    a. Yes
    b. No

11. **Is this a certified organic herd?**
    a. Yes
    b. No

12. **What is the normal calving season?**
    a. Year-round
    b. Spring
    c. Fall
    d. Other (please specify) __________________

13. Do you utilize artificial insemination for cattle breeding? 1. Yes 2. No

14. Do you utilize embryo transfer? 1. Yes 2. No

15. Do you hunt or allow hunting on your property? 1. Yes 2. No
   a. **If yes, please specify which species? ____________________________________**
   b. **If deer hunting occurs, are gut piles removed or left in the field? 1. Yes 2. No**

16. Are cows tested for *Neospora caninum*?
   a. Yes, all cows
   b. Yes, only cows that abort
   c. No
   d. Other (please specify)______________

17. Are placentas and/or aborted tissues disposed of?
   a. Yes
      i. If yes, how and where?____________________
   b. Sometimes
      i. Explain: _________________________
   c. No

18. Are dead animals disposed of? (This includes any animal found dead on the farm.)
   a. Yes
      i. If yes, how and where?____________________
   b. Sometimes
      i. Explain: _________________________
   c. No

19. Is colostrum pooled?
   a. Yes
   b. Sometimes
   c. No

20. If colostrum is not pooled, are calves allowed to suckle colostrum?
   a. Yes
   b. No

Section C: Biosecurity

21. When did you last purchase cattle? Date and origin of cattle acquired?

Last purchase (month/year): ___________________

Source of Cattle to herd
   a. No cattle introduced including bulls

b. Known source of cattle (please specify name and location) ________________________
c. Dealer, sale barn (please specify name and location) _____________________
d. Unknown

Number of cattle introduced to herd during the last purchase: __________________

What is/are the source(s) of replacement heifers on your farm? (please specify name and location) _________________

22. Before cattle are purchased, what vaccination and/or health requirements do you have?

---

23. Does this farm have fence line contact with other neighboring cattle herds?

1. Yes   # Herds: __________
2. No

24. Is there a house located on the farm property?

1. Yes   Distance from main facilities? ___________________________
2. No

18. Is there a dog(s) located on the premises?

1. Yes   2. No

If Yes:

How many dogs are present? __________________

What is the age of each dog? __________________

When was each dog acquired? ____

Do you breed dogs or raise litters on the premises? 1. Yes 2. No

Does the dog(s) ever have access to the cattle facilities? 1. Yes 2. No

Does the dog(s) ever have access to the cattle feed? 1. Yes 2. No

Does the dog(s) ever have access to aborted materials (fetuses, uterine discharge, placentas)?

1. Yes   2. No

If No:

Has there been an outside dog(s) located on the premises within the past 10 years?

1. Yes 2. No 3. Unknown

19. Are there stray dogs located on the farm?

1. Yes 2. No

If No:

Have there been stray dogs located on the farm in the past 10 years?

1. Yes 2. No 3. Unknown

21. What type of fencing is used on the farm?

1. Barbed Wire
2. Electrified wire
3. Combination barbed wire/electrified
4. Woven wire
5. Other: ____________________

25. What is the minimum fence height (perimeter fence) on the farm?

1. Less than 6 feet
2. 6-9 feet
3. At least 10 feet

26. Indicate which wildlife species you have observed on or in close proximity to (less than ½ mile) the farm and how frequently you observe them (i.e. daily, weekly, monthly, etc.).

☐ **birds (house sparrows) ______________________**
☐ **wild cats __________________**
☐ **foxes _____________________**
☐ **coyotes ___________________**
☐ **wolves ___________________**
☐**deer ______________________**
☐ **rodents ___________________**
☐ **skunks ____________________**
☐ **raccoons __________________**
☐ **other _____________**

27. Have wildlife species interfered with your cattle or cattle management practices in the past? (e.g. depredation of cattle, access to feed, etc.)

1. Yes 2. No

If Yes:

Please describe the situation:

____

28. Do you utilize any pest management strategies on the farm for rodent or wildlife control (i.e. baits, traps, etc.)? 1. Yes 2. No

a. **If yes, please explain:** ______________________________________

Section D: Herd Health

29. What cattle vaccines do you use? ______________________________

30. Have any of your cattle attended cattle shows in the past 2 years?

a. Yes b. No

31. Do any of the cattle have a history of any of the following?

- ☐ **BVDV**
- ☐ **BHV-1**
- ☐ **BLV**
- ☐ **high somatic cell count**
- ☐ **retained placental tissues**

32. What is the protocol for the management of sick animals? (e.g. isolation from herd, calving pens used to hospitalize sick animals, etc.)

____

33. Do you use birthing pens? 1. Yes 2. No

a. **If yes, how often are pens cleaned?** ____________

34. What is the average herd conception rate? _________

35. What is the average herd birth rate? __________

36. Does the farm have a history of abortions in cattle?

a. Yes b. No

If Yes:

Were the abortion(s) ever positively attributed to *Neospora caninum* infection?

a. Yes b. No

What are the average ages of cattle that have aborted in the past?

- [ ] **first calf heifers**
- [ ] **young adult cows (2-3 births)**
- [ ] **older adult cows (more than 3 births)**

Is there a time of year they occur more frequently?

a. Yes b. No

If Yes:

i. When? ________________________

31. Is there a history of cattle giving birth to weak calves?

a. Yes b. No

A. General Farm Characteristics		
**Risk Factor**	**Scoring Guidelines**	**Score**
1. Feed Storage	Outside, unfenced, uncovered<br><br>Outside, fenced, uncovered	High<br><br>Moderate
	Outside, unfenced, covered<br><br>Outside, fenced, covered	Moderate<br><br>Moderate
	In an open building<br><br>In an enclosed building	Moderate<br><br>Low
2. Water	Stream, lake, pond<br><br>Well water (non-potable)<br><br>Well water (potable)<br><br>Public Water supply	High<br><br>Moderate<br><br>Low<br><br>Low
**B. Facilities and Management**		
**Risk Factor**	**Scoring Guidelines**	**Score**
3. Calving season	Spring<br><br>Fall<br><br>Year-round	
4. *N. caninum* testing protocols	No testing performed<br><br>Testing of cows that abort, positive are not culled<br><br>Testing of cows that abort, positive are culled<br><br>All cows are tested, positive are culled	High<br><br>High<br><br>Moderate<br><br>Low
5. Placenta, aborted tissue, and dead stock disposal	Left outside in the open<br><br>Removed and buried to prevent predation<br><br>Composted – accessible<br><br>Composted - fenced<br><br>Removed from premises	High<br><br>Moderate<br><br>Moderate<br><br>Low<br><br>Low
**Section C: Biosecurity**		
**Risk Factor**	**Scoring Guidelines**	**Score**
6. Source of cattle introduced to herd	Cattle from herds with unknown *N. caninum* status	High

	and unknown reproductive status<br><br>Only cattle from herds with good reproductive status and unknown *N. caninum* status<br><br>Only cattle from *N. caninum* free herds	 <br>Moderate<br><br><br>Low
7. Owned and/or stray dogs on premises	Dog(s) have free or occasional access to cattle housing and/or feed storage areas<br><br>Dog(s) are located on premises but have no access to cattle housing and/or feed storage areas<br><br>There are no dogs allowed on the premises	High<br><br><br>Moderate<br><br><br>Low
8. Wild canids on farm (coyotes, fox, wolf)	Wild canids are frequently seen on the premises in cattle housing and/or feed storage areas<br><br>Wild canids are seen on the premises but have no access to cattle housing and/or feed storage areas<br><br>There are no wild canids seen on the premises	High<br><br><br>Moderate<br><br><br>Low
**Section D: Herd Health**		
9. Use of isolation pens for birthing	Isolation pens are often used as birthing pens<br><br>Isolation pens are sometimes used as birthing pens<br><br>Isolation pens are never used as birthing pens	High<br><br>Moderate<br><br><br>Low
10. History of abortion	Yes, history of abortions attributed to *N. caninum*<br><br>Yes, history of abortions due to unknown cause<br><br>Yes, history of abortions due to a known cause other than *N.*	High<br><br>High<br><br>Moderate

	caninum<br><br>No history of abortions	 <br>Low
